# Upper extremity training followed by lower extremity training with a brain-computer interface rehabilitation system

**DOI:** 10.3389/fnins.2024.1346607

**Published:** 2024-03-04

**Authors:** Sebastian Sieghartsleitner, Marc Sebastián-Romagosa, Woosang Cho, Johannes Grünwald, Rupert Ortner, Josef Scharinger, Kyousuke Kamada, Christoph Guger

**Affiliations:** ^1^g.tec Medical Engineering GmbH, Schiedlberg, Austria; ^2^Institute of Computational Perception, Johannes Kepler University, Linz, Austria; ^3^g.tec Medical Engineering Spain S.L., Barcelona, Spain; ^4^Department of Neurosurgery, Megumino Hospital, Eniwa, Japan

**Keywords:** brain-computer interface, motor imagery, EEG, rehabilitation, upper extremity, lower extremity, motor function, stroke

## Abstract

**Introduction:**

Brain-computer interfaces (BCIs) based on functional electrical stimulation have been used for upper extremity motor rehabilitation after stroke. However, little is known about their efficacy for multiple BCI treatments. In this study, 19 stroke patients participated in 25 upper extremity followed by 25 lower extremity BCI training sessions.

**Methods:**

Patients’ functional state was assessed using two sets of clinical scales for the two BCI treatments. The Upper Extremity Fugl-Meyer Assessment (FMA-UE) and the 10-Meter Walk Test (10MWT) were the primary outcome measures for the upper and lower extremity BCI treatments, respectively.

**Results:**

Patients’ motor function as assessed by the FMA-UE improved by an average of 4.2 points (*p* < 0.001) following upper extremity BCI treatment. In addition, improvements in activities of daily living and clinically relevant improvements in hand and finger spasticity were observed. Patients showed further improvements after the lower extremity BCI treatment, with walking speed as measured by the 10MWT increasing by 0.15 m/s (*p* = 0.001), reflecting a substantial meaningful change. Furthermore, a clinically relevant improvement in ankle spasticity and balance and mobility were observed.

**Discussion:**

The results of the current study provide evidence that both upper and lower extremity BCI treatments, as well as their combination, are effective in facilitating functional improvements after stroke. In addition, and most importantly improvements did not stop after the first 25 upper extremity BCI sessions.

## 1 Introduction

Globally, stroke is a leading cause of mortality and disability in adults 50 years and older (Feigin et al., 2021). Fortunately, stroke prevalence and mortality rates have decreased in recent decades due to advances in prevention, as well as treatment of acute stroke (Vos et al., 2020). However, absolute numbers of stroke deaths and healthy life years lost are still rising, given population growth and higher life expectancies (Feigin et al., 2021). Substantial motor recovery can occur in the first weeks after stroke (Kwakkel et al., 2004). Nonetheless, two-thirds of stroke survivors recover insufficient upper extremity (UE) function to perform activities of daily living (ALDs) (Jørgensen et al., 1995b; Kwakkel et al., 2003, 2004; Krakauer, 2006). Similarly, 54 to 80% of stroke survivors are affected by gait disorders (Perry et al., 1995; Jørgensen et al., 1995a; Cho et al., 2014), which strongly impacts survivors’ autonomy and disability (Duarte et al., 2010; Jarvis et al., 2019; Tasseel-Ponche et al., 2022).

Brain-computer interfaces (BCIs) utilize users’ brain activity to control external devices without the need of actual movements (Wolpaw et al., 2002; Belkacem et al., 2020). This brain activity can be recorded using electroencephalography (EEG), electrocorticography, stereo electroencephalography, functional near-infrared spectroscopy (fNIRS) or functional magnetic resonance imaging (fMRI), with EEG being used the most (Orban et al., 2022; Islam and Rastegarnia, 2023). Recently, BCIs using EEG have emerged as a promising technology for UE motor rehabilitation after stroke (Mane et al., 2022). In this context, BCIs establish a closed-loop system between the user and the external device(s). This interaction between BCI and user is facilitated by providing meaningful real-time feedback in response to motor-related neural activity. The users, themselves, perform motor execution, movement attempts or motor imagery (MI), with MI being the mental rehearsal of a movement. Importantly, all three strategies are accompanied by event-related desynchronization (ERD) and synchronization (ERS), which reflect decreases and increases in oscillatory power (Pfurtscheller and Lopes da Silva, 1999; Pfurtscheller et al., 2006; Miller et al., 2010). Different external devices (e.g., robotics, arm orthoses, visual feedback, functional electrical stimulation (FES)) can be used to provide feedback to the user, with devices which provide proprioceptive feedback likely being more effective than just visual feedback (Ono et al., 2014; Bai et al., 2020). Specifically, BCIs which trigger FES (BCI-FES) are thought to be the most effective (Bai et al., 2020). Meta-analyses have shown that BCIs for UE motor rehabilitation can improve UE motor function (Bai et al., 2020; Kruse et al., 2020). However, less is known for lower extremity (LE) motor rehabilitation. Recent BCI studies based on movement-related cortical potential (Mrachacz-Kersting et al., 2016), BCI-FES (Chung et al., 2020; Sebastián-Romagosa et al., 2023) and functional near-infrared spectroscopy-mediated neurofeedback (Mihara et al., 2021) showed improvements in gait performance. Sebastián-Romagosa et al. (2023) showed a walking speed improvement of 0.19 m/s across 25 sessions. However, the impact of multiple BCI treatments on stroke patients’ functional state has to date not been investigated.

We here present a study using a BCI system based on MI, FES and a realistic 3D avatar for visual feedback. The same group of stroke patients trained with this BCI system for 25 UE followed by 25 LE therapy sessions. We investigate the BCI system’s efficacy in facilitating UE and LE motor function improvements and provide novel insights regarding the effects of two consecutive BCI treatments, as well as patients’ BCI performance during UE and LE BCI training.

## 2 Materials and methods

### 2.1 Participants and study design

The current study was approved by the Ethikkommission des Landes Oberösterreich in Austria (Nr. 1,126/2020 and #D-42-17) and the Bundesamt für Sicherheit im Gesundheitswesen (clinical trial number 101210314) in Austria. Participants provided written informed consent.

**Figure 1:**
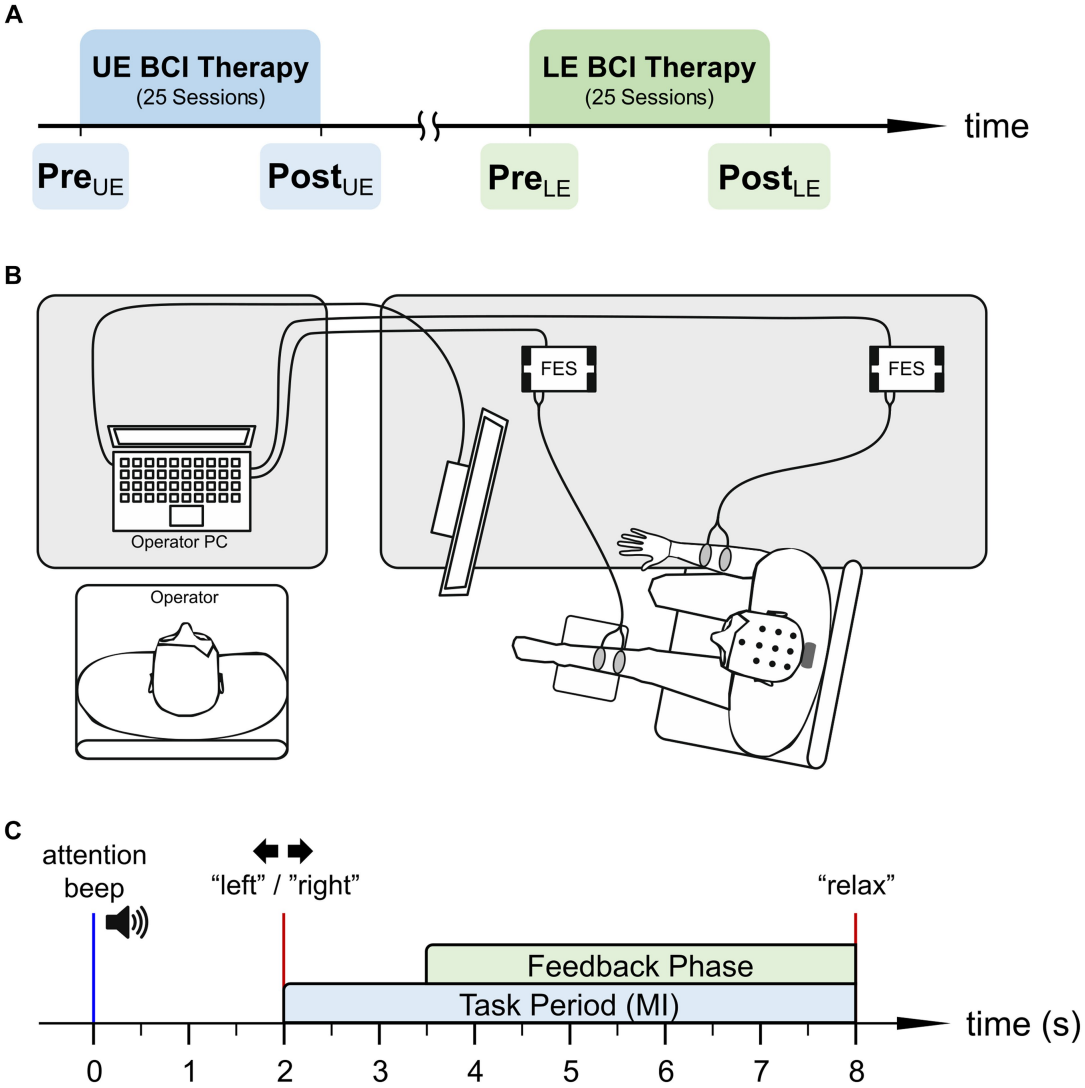
**(A)** Time course of upper and lower extremity (UE and LE) BCI treatment and the respective pre- and post-assessments. **(B)** Schematic of the BCI system setup used for lower extremity treatment including operator and patient. **(C)** Time course of a single motor imagery (MI) trial.

Nineteen stroke patients with UE and LE hemiparesis participated in two BCI treatments, which were performed in the rehabilitation center recoveriX Gym in Schiedlberg, Austria. During these BCI treatments patients did not participate in other motor rehabilitation therapies. Each treatment lasted up to 3 months with 2 to 3 sessions per week and 25 sessions in total. The time between the two BCI treatments varied across patients as patients participated in an UE study followed by a LE study. In this publication we analyze the evolution of these patients through the two different BCI treatments. Pre- and post-assessments were performed before and after the UE and LE BCI treatment, respectively (see Figure 1A). During these assessments patients’ functional state was evaluated using clinical scales.

### 2.2 BCI system description

Patients trained with a BCI system based on MI, a realistic 3D avatar for visual feedback and FES (recoveriX, g.tec Medical Engineering GmbH, Austria). During the therapies patients wore wireless EEG caps appropriate for their head size with 16 active electrodes (g.Nautilus PRO, g.tec Medical Engineering GmbH, Austria) covering the sensorimotor cortex (i.e., FC3, FCZ, FC4, C5, C3, C1, CZ, C2, C4, C6, CP3, CP1, CPZ CP2, CP4, and PZ according to the international 10/20 system). The reference electrode was mounted on the right earlobe and the ground electrode was placed at AFZ.

During the upper extremity therapy, patients sat in a chair in front of a computer screen with both forearms resting on the desk and surface FES electrode pairs attached to their left and right wrist extensors. In contrast, during the lower extremity therapy, patients sat almost parallel to the desk with the affected leg slightly elevated on a leg rest (see Figure 1B). In this case, the two surface FES electrodes were attached to the wrist dorsiflexor of the healthy side and the foot dorsiflexor of the paretic side, respectively. For both the hand and foot dorsiflexors, the stimulation frequency and the pulse width of the FES stimulation (g.Estim FES, g.tec Medical Engineering GmbH, Austria) were set to 50 Hz and 300–400 μs, respectively. The stimulation current was adjusted during each session to facilitate (i) optimal passive movement without pain for patients with mild or moderate muscle spasm, or (ii) muscle contraction in the target muscle of the paretic side for patients with severe muscle spasm (Sebastián-Romagosa et al., 2020b).

### 2.3 Motor imagery and feedback

Figure 1C shows the time course of a single MI trial (i.e., one repetition). Each trial started with an attention beep, indicating to the patients that soon the MI task will begin. Two seconds later patients were instructed to either perform a left- or right-side MI. This instruction was shown as an arrow on the patient’s monitor and additionally provided via headphones as a spoken word (“left”/“right”) in the patients’ native language. The patients were instructed to imagine a dorsiflexion of the respective side during the MI task, until the spoken word “relax” indicated the end of the trial. The feedback phase started 3.5 s after the attention beep. During this phase, the BCI provided synchronous visual and proprioceptive FES feedback to the patient, if the classified MI side matches the instruction. Patients were instructed to keep performing MI during feedback as this additionally increases cortical activation (Reynolds et al., 2015).

A detailed description of feedback presentation and signal processing can be found in Irimia et al. (2018), Sebastián-Romagosa et al. (2020a) and Sebastián-Romagosa et al. (2023). Nonetheless, it is briefly described here. Each BCI therapy session is made up of three runs, each lasting roughly 15 min and containing 40 MI trials per side. The first run of each session is used as calibration run to obtain the EEG data necessary to train the classifier. In order to obtain the trained classifier first EEG data were band pass filtered (8–30 Hz), followed by application of common spatial patterns (CSP), which are used to maximize the variance between the two classes (i.e., left and right MI) (Blankertz et al., 2008). Following the application of the CSP the log-transformed variance was computed and finally input to the linear discriminant analysis (LDA), which was used as classifier. The feedback provided to the patients was updated every 200 ms.

### 2.4 Functional and behavioral assessment

Two different sets of clinical scales were used for the assessments during the UE and LE treatments.

For the UE assessments, three clinical scales were used: Fugl-Meyer Assessment for the upper extremity (FMA-UE), Barthel Index (BI), and modified Ashworth Scale (MAS) (Mahoney and Barthel, 1965; Fugl-Meyer et al., 1975; Ansari et al., 2008) for the paretic wrist and fingers. These scales are frequently used in the context of stroke, with FMA-UE and BI having excellent (Duncan et al., 1983; Sanford et al., 1993; Duffy et al., 2013) and MAS having moderate to high (Gregson et al., 1999; Ansari et al., 2008) intra- and interrater reliability. For UE assessment, we used FMA-UE as the primary outcome measure, as it is recommended for evaluating upper extremity motor function after stroke (Gladstone et al., 2002; Bushnell et al., 2015).

For the LE assessments, four clinical scales were used: The 10-Meter Walk test (10MWT), BI, Timed Up and Go (TUG) test and MAS for the paretic knee and ankle (Podsiadlo and Richardson, 1991). Although functionally unrelated the FMA-UE was also performed during the lower extremity assessments for monitoring purposes. The 10MWT and TUG test have excellent test–retest, as well as intra- and interrater reliability (Collen et al., 1990; Flansbjer et al., 2005; Lyders Johansen et al., 2016; Cheng et al., 2021). For LE assessment, we used the 10MWT as the primary outcome measure, as it is the gold standard for short-distance walking speed in stroke patients (Cheng et al., 2021). Walking speed after stroke is an important measure as it relates to vital status and gait ability (Mayo et al., 1999; Fulk and Echternach, 2008; Fritz and Lusardi, 2009; Chiu et al., 2012; Kwakkel et al., 2017). Note that the 10MWT may be performed and reported in a multitude of ways. In this study, patients were instructed to walk 10 meters at a fast but safe speed. The duration t it took them to walk from meter 2 to meter 8 (i.e., a distance of 6 meters) was measured with a stopwatch. All patients repeated the 10MWT three times per assessment, yielding three durations $t_{1}$ , $t_{2}$ , and $t_{3}$ . Finally, we used the minimum duration to estimate the maximum walking speed.

### 2.5 Statistical analysis of clinical scales

We used MATLAB (The MathWorks Inc., United States) to perform our statistical analyses. Specifically, we used statistical tests to analyze the changes in each clinical scale between pre- and post-assessments for the upper and lower extremity BCI treatment, respectively. Additionally, we investigated the between BCI treatment changes (UE_Post_ vs. LE_Pre_) and across BCI treatment changes (UE_Pre_ vs. LE_Post_) for the BI and FMA-UE, as they are common two both sets of clinical scales (see Section 2.4).

The appropriate statistical test and descriptive statistics were chosen based on the normality (i.e., gaussianity) of the data. Specifically, we assessed normality of the differences (e.g., Post – Pre) using the Shapiro–Wilk test at a significance level of α = 0.05 (Yap and Sim, 2011). If the Shapiro–Wilk test rejected the null hypothesis, meaning the data are likely not normally distributed, a Wilcoxon signed-rank test was used. Otherwise, a paired *t*-test was used. Descriptive statistics are reported as mean and standard deviation (SD) for normally distributed data, and median and inter-quartile range (IQR; 25th and 75th percentile) otherwise.

As multiple statistical tests were carried out, we corrected the obtained *p*-values for multiplicity (i.e., multiple hypothesis testing) using the Benjamini-Hochberg (i.e., false discovery rate) procedure (Benjamini and Hochberg, 1995).

### 2.6 BCI performance

The BCI performance was defined as the mean classification accuracy during the feedback phase, which lasts from 3.5 to 8 s in each trial (see Figure 1C). These classification accuracies were obtained offline using 10 repetitions of a 10-fold cross-validation for each BCI session (i.e., 120 trials per class), with the same processing framework as online [see Section 2.3, as well as Sebastián-Romagosa et al. (2023)]. Following the extraction of the BCI performance we performed two separate analyses for the UE and LE treatment:

In the first analysis, we aimed to gain insights regarding MI learning effects. Therefore, we used the appropriate paired statistical test to assess differences in BCI performance between early and late therapy sessions. Specifically, we compared the median BCI performance during the first 5 and last 5 sessions.

In the second analysis, we focused on the relationship in BCI performance during the UE and LE treatment. Specifically, we analyzed the difference in median BCI performance during the UE and LE treatment using the appropriate paired statistical test, as well as their relationship using Spearman’s rank correlation.

The *p*-values obtained in these two analyses were corrected for multiplicity using the Benjamini-Hochberg procedure.

## 3 Results

Table 1 shows the baseline characteristics for the UE and LE treatments. Patients’ mean age was 53.1 years with 8 and 11 of them being female and male, respectively. The median time since stroke was 23.6 months and ranged from 3 to 376 months, with one out of 19 patients being in the subacute phase. Sixteen patients suffered an ischemic stroke with 10, 3 and 3 patients having subcortical, cortical+subcortical and brainstem lesions, respectively. The remaining three patients suffered a hemorrhagic stroke with cortical+subcortical lesions. This classification with respect to lesion location was performed using medical records, as well as medical imaging. The median FMA-UE score was 19.0 points before the UE treatment with 15, 1 and 3 patients being severely, moderately, and mildly impaired, respectively (Woytowicz et al., 2017). The median time between treatments was 7.4 months and the median walking speed was 1.2 m/s before the LE treatment.

**Table 1:** Baseline characteristics of patient sample.

	Upper extremity	Lower extremity
Age (years)	53.1 (16.5)	54.4 (16.0)
Female/male	8/11	8/11
Time since stroke (months)	23.6 [12.5; 56.5]	36.8 [23.2; 79.6]
Primary outcome measure^*^	19.0 [13.3; 26.8]	1.2 [0.4; 1.2]
Time between treatments (months)	–	7.4 [3.6; 16.8]

Descriptive statistics reported in mean (SD) or median [IQR]. SD and IQR are the standard deviation and the inter-quartile range, respectively. ^*^FMA-UE (points) for Upper extremity, 10MWT (m/s) for Lower extremity.

### 3.1 Upper extremity and lower extremity BCI treatment

It is of course interesting to see the improvement after each of the BCI treatments. Table 2 shows the baseline (Pre) and change (Post – Pre) in clinical scales for the UE treatment and LE treatment, including the *p*-values.

**Table 2:** Functional improvement after UE and LE BCI treatment.

BCI treatment	Clinical scale	*n*	Pre	ΔPost-Pre	*p*	CRT reached
Upper extremity	FMA-UE	19	19.0 [13.2; 26.8]	4.2 (4.0)	<0.001	Yes
	BI	19	85.0 [67.5; 93.8]	5.0 [0.0; 5.0]	<0.001	–
	MAS wrist	19	2.0 [1.5; 3.0]	−1.0 [−1.0; 0.0]	<0.001	Yes
	MAS finger	17	2.0 [2.0; 3.0]	−1.0 [−1.1; −0.4]	<0.001	Yes
Lower extremity	10MWT (m/s)	19	1.16 [0.43; 1.23]	0.15 (0.15)	0.001	Yes
	BI	18	87.5 [80.0; 90.0]	0.0 [0.0; 5.0]	0.049	–
	FMA-UE	19	18.0 [13.5; 29.8]	3.3 (3.0)	<0.001	No
	TUG (s)	19	15.4 [13.0; 38.8]	−2.7 [−14.5; −1.8]	<0.001	Yes
	MAS knee	7	1.5 [1.0; 1.9]	−0.5 [−1.0; 0.0]	0.051	Yes
	MAS ankle	14	2.2 [1.0; 3.0]	−0.5 [−1.0; 0.0]	0.011	Yes

Units of clinical scales reflect points, except for 10MWT and TUG which measure m/s and seconds, respectively. Descriptive statistics reported in mean (SD) or median [IQR]. *p*-values are corrected for multiplicity using the Benjamini-Hochberg procedure. SD, standard deviation. IQR, inter-quartile range. CRT, Clinically relevant threshold. A dash (“-“) indicates that no CRT definition is available.

**Figure 2:**
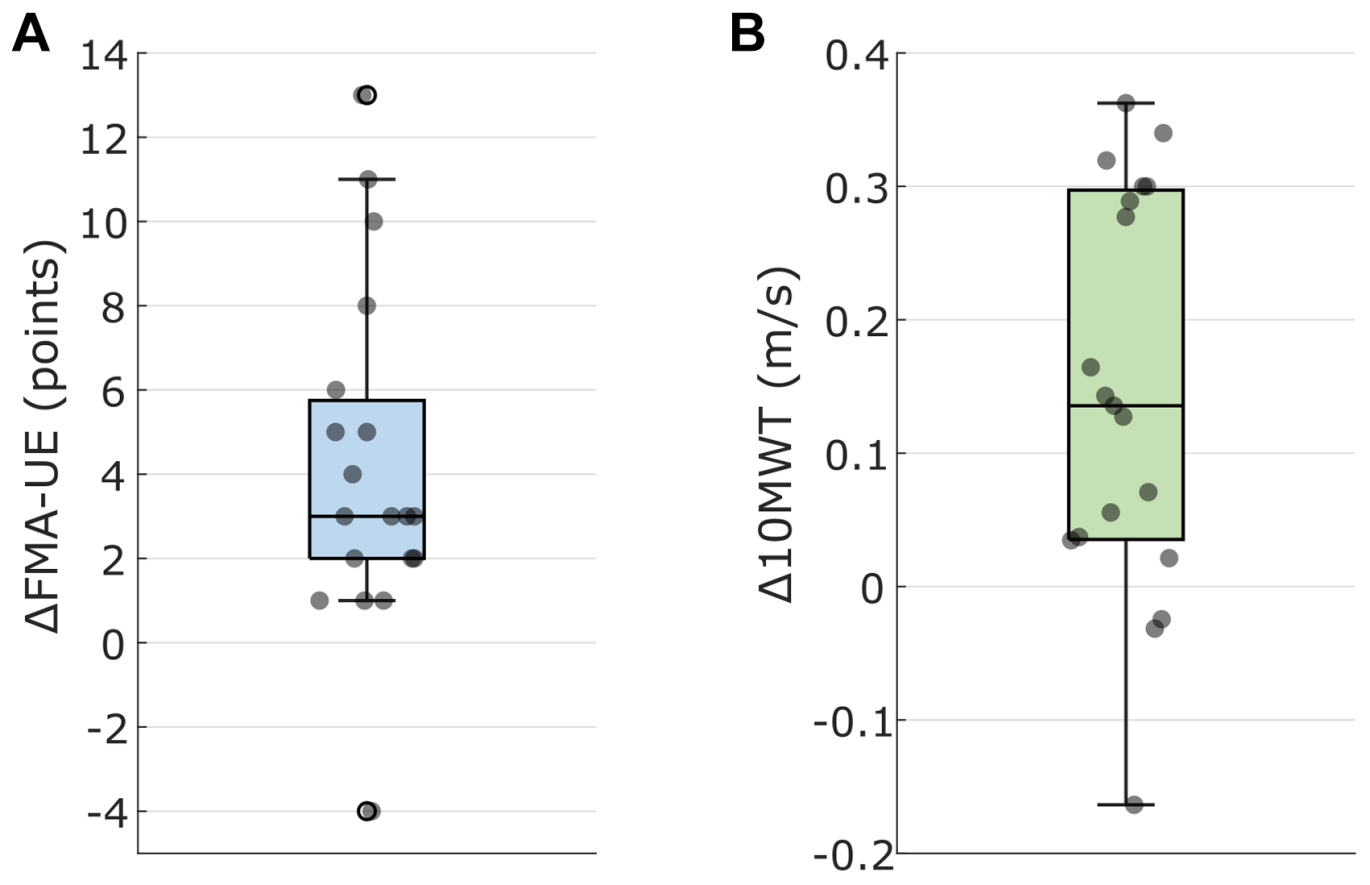
**(A)** Change in Upper Extremity Fugl-Meyer Assessment (FMA-UE) after upper extremity BCI treatment (UE_Post_ – UE_Pre_). Individual data points reflect patients. **(B)** Change in 10-Meter Walk Test (10MWT) velocity after the lower extremity BCI treatment (LE_Post_ – LE_Pre_). Individual data points reflect patients.

The FMA-UE increased by 4.2 (4.0) points (*p* < 0.001), reflecting an improvement in UE motor function (see Figure 2A). Patients’ BI improved by 5.0 [0.0; 5.0] points (*p* < 0.001) indicating improvements in activities of daily living. Patients’ wrist and finger spasticity decreased by −1.0 [−1.0; 0.0] points (*p* < 0.001), respectively. Note that the MAS assesses spasticity on a scale from 0 to 4 points with 0 points indicating no increase in muscle tone and 4 points indicating rigidness in flexion or extension. For MAS finger, two patients were excluded from the statistical analysis as they had no increase in muscle tone in both their pre- and post-assessment.

Patients improved in their gait speed by 0.15 (0.15) m/s according to the 10MWT (*p* = 0.001) (see Figure 2B). Patients’ BI increased by 0.0 [0.0; 5.0] points (*p* = 0.049). One patient reached a BI of 100 points in both pre- and post-assessment and was thus excluded from the statistical analysis. Interestingly, patients FMA-UE also increased by 3.3 (3.0) points (*p* < 0.001). Patients improved in their TUG test by −2.7 [−14.5; −1.8] seconds (*p* < 0.001), representing a relative time decrease of −24.5 (15.0)% in mean (SD). Patients’ ankle spasticity decreased significantly by −0.5 [−1.0; 0.0] points (*p* = 0.011). Patients without increase in muscle tone were excluded from the statistical analysis for MAS and therefore we had only 7 (knee) and 14 (ankle) patients with MAS.

The last column of Table 2 shows whether the clinically relevant thresholds (e.g., clinical important difference, meaningful change) were reached for the respective clinical scale. For the FMA-UE the clinically relevant thresholds are 3.5 points and 4.25 to 7.25 points depending on whether chronic stroke patients are severely or moderately to mildly impaired (Page et al., 2012; Barden et al., 2023). These two thresholds were reached for the respective patient groups in the current study. Furthermore, the thresholds were also reached for the MAS (Mangold et al., 2009; Chen et al., 2020), 10MWT (Perera et al., 2006) and TUG (Flansbjer et al., 2005). For BI, as described and recommended by Quinn et al. (2011), we did not have a definition of the clinically important difference.

Between the UE and LE treatment, the BI and the FMA-UE did not change significantly. Across both UE and LE treatment (LE_Post_ – UE_Pre_) FMA-UE increased by 4.8 (5.5) points (*p* = 0.002) and BI increased by 8.4 (11.1) points (*p* = 0.007).

### 3.2 BCI performance

**Figure 3:**
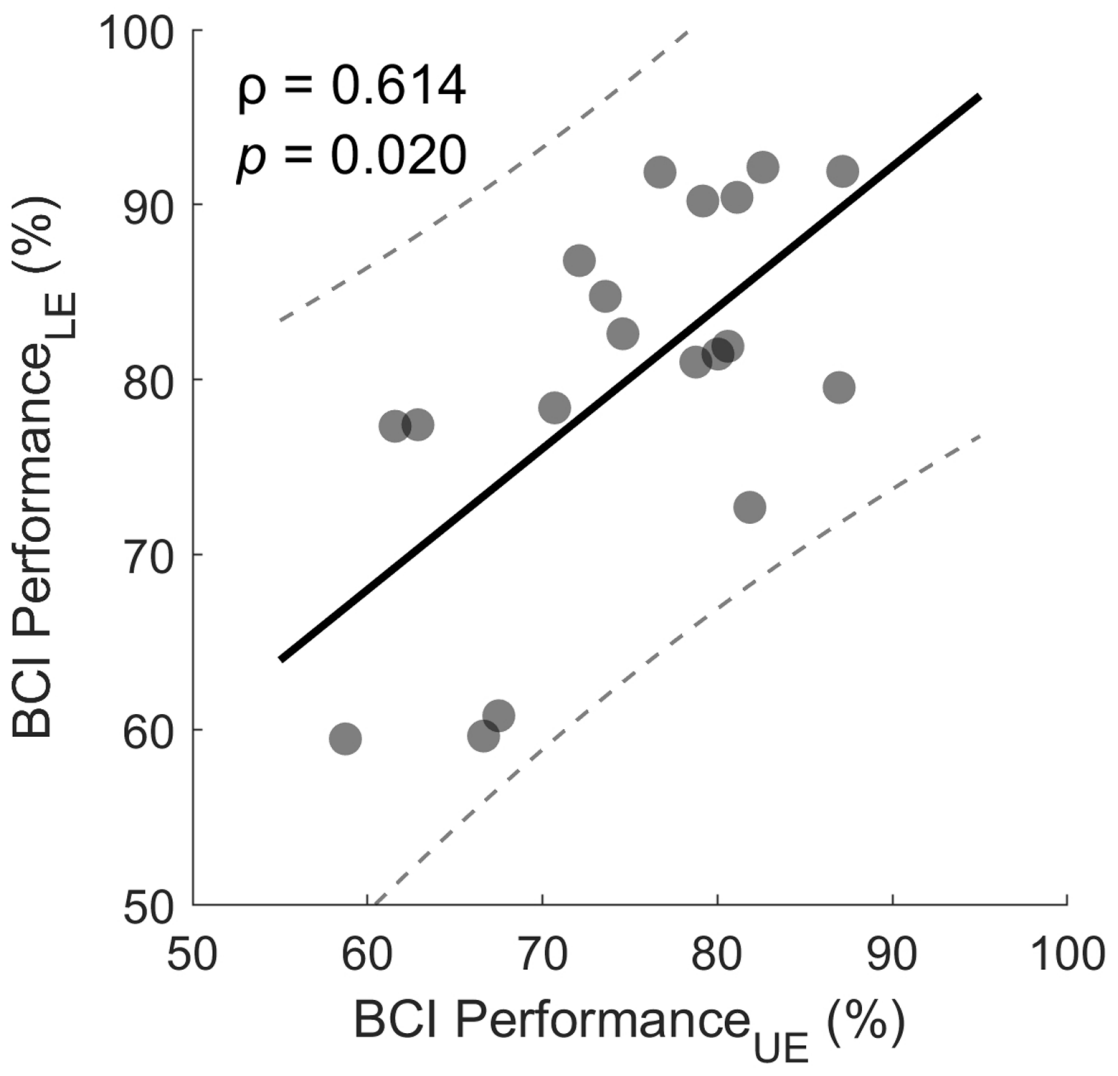
Correlation between UE and LE BCI performance. Each dot represents the median performance of one subject over all sessions. The solid line indicates straight line fit and the two dashed lines indicate the 95% prediction interval.

Table 3 shows the BCI performance across patients during the UE and LE treatment. BCI performance was generally good and during the UE treatment patients were able to improve their BCI performance by 3.4 (5.2) % between early and late sessions (*p* = 0.020). In contrast, this was not the case for the LE treatment (*p* = 0.102). However, patients’ median BCI performance across all sessions was generally greater by 5.1 (8.2) % in the LE compared to the UE treatment (paired t-test t(18) = 2.7, *p* = 0.020). Finally, BCI performances in UE and LE were moderately correlated according to Spearman’s rank correlation (ρ = 0.614, *p* = 0.020, see Figure 3).

**Table 3:** BCI performance (%) for upper and lower extremity BCI treatment in the first and last 5 therapy sessions.

BCI treatment	BCI performance (%)			*p*
	Early sessions	Late sessions	ΔLate-Early	
Upper extremity	72.5 (8.8)	75.9 (9.2)	3.4 (5.2)	0.0195
Lower extremity	79.1 (11.3)	81.9 (10.7)	2.8 (7.1)	0.1016

Descriptive statistics reported in mean and standard deviation in braces. *p*-values are corrected for multiplicity using the Benjamini-Hochberg procedure.

## 4 Discussion

In the present study we investigated the efficacy of UE and LE BCI treatment in stroke patients, as well as the effects of two consecutive BCI treatments (i.e., UE followed by LE BCI treatment). Patients UE motor function, activities of daily living and hand as well as finger spasticity improved after the UE treatment. After the LE treatment further improvements in activities of daily living and UE motor function, as well as improvements in walking speed, functional mobility and ankle spasticity were observed. Taken together, these results show that the positive effects did not stop after the first 25 therapy sessions and that patients benefited from both UE and LE BCI treatments.

### 4.1 Upper extremity BCI treatment

The primary outcome measure for the UE BCI treatment was the FMA-UE which assess UE motor function. Patients improved significantly in their UE motor function by 4.2 points on average after the UE treatment. The percentage improvement in FMA-UE was 22% on average and 18 out of the 19 patients showed an improvement in FMA-UE score. However, it is important to point out that the patient who did not improve according to FMA-UE still improved in ADLs, as well as wrist spasticity. Important is as well that the median FMA-UE score was 19 which indicates that patients tended to be severely impaired. In fact, 15 patients were severely impaired, one patient was moderately impaired and 3 patients were mildly impaired and therefore an improvement of 4.2 points for mostly severely impaired patients is noteworthy. The highest improvements are typically reached in moderately impaired patients, because there is no ceiling effect like with mildly impaired patients and spasticity is not as bad as in severely impaired patients. Nonetheless, both severely and moderately to minimally impaired patients reached the clinically important difference thresholds for the FMA-UE.

Patients’ baseline BI was relatively high at 85.0 points on median and further increased by 5 points, representing a significant increase in performance in ADLs. In general, 13 out of the 19 patients showed improvements in BI. Interesting is as well that the BI is relatively high even though patients tended to have severe UE impairments, showing again that these patients learned to compensate most of the daily activities with the healthy hand and arm.

Improvements in wrist and finger spasticity play an essential role as they allow for improvements in fine and gross motoric skills. Patients’ wrist and finger spasticity decreased by −1.0 points on median, respectively. Importantly, these reductions in spasticity exceed clinically important difference. Thirteen out of the 19 patients show improvements in wrist and finger spasticity, respectively. Two patients did not have any spasticity in the pre-assessment and post-assessment which is as well important that the therapy did not negatively affect it. In fact, spasticity did not increase for any of the patients in the current study.

The improvements in FMA-UE observed in the present study are similar to ones observed in BCI studies with similar baseline impairment and time since stroke: 3.4 points by Ramos-Murguialday et al. (2013), 6.6 points by Biasiucci et al. (2018), 4.7 points by Sebastián-Romagosa et al. (2020a) and 3.5 points by Miao et al. (2020). Nonetheless, there are noteworthy differences between these BCI studies. Specifically, Ramos-Murguialday et al. (2013) and Biasiucci et al. (2018) instructed patients to perform motor executions/attempts with their affected side, whereas here patients are performing MI of both their affected and healthy hand. While such bilateral training is more difficult it is, however, more engaging and leads to a more active therapy. Additionally, bilateral training was found to be more effective in improving motor impairment in UE stroke patients (Chen et al., 2019). Finally, Ramos-Murguialday et al. (2013) employed a hand orthosis for finger extension as feedback mechanism.

The present study consisted of mostly severely impaired patients, who are known to have slower and less functional recovery compared to moderately impaired patients. These severely impaired patients were, however, motivated enough to do both the UE and LE treatment to achieve greater improvements.

Before starting the LE BCI treatment, we assessed the functional state of the patients again. Between UE and LE treatment no significant changes were found in BI and FMA-UE, indicating that on a group level patients’ independence in ADLs and UE motor function stayed reasonably stable across a median time between treatments of 7.4 months. After the UE treatment patients started to move their UE more in daily life and therefore muscles and movements were trained even in the time after the treatment and therefore, they did not decline in these 7.4 months.

### 4.2 Lower extremity BCI treatment

The primary outcome measure for the LE BCI treatment was the 10MWT, reflecting walking speed in m/s during the middle 6 meters of the 10MWT. After the LE treatment patients were able to walk 0.15 m/s faster on average, representing a percentage improvement of 23%. The small and substantial meaningful changes for the 10MWT are estimated to be 0.05 and 0.10 m/s which were exceeded by 13 and 11 patients, respectively (Perera et al., 2006). Sixteen out of 19 patients improved in their walking speed. However, all three apparent non-responders improved in their TUG performance with one of them even improving by 40%. Furthermore, in the same subgroup of patients, ankle spasticity decreased if it was present in the pre-assessment. Finally, two out of the three patients improved in ADLs with the other one staying at 95 points in BI.

Patient’s baseline BI was 90.0 points on median and further improved according to the statistical test. However, the numerical median change was found to be zero. Eight out of the 19 patients improved in their ADLs according to their BI scores. Note that baseline BI was already close to the maximum value of 100 points and that the BI is quantified in steps of 5 points. A clinical important decrease in ankle spasticity of −0.5 points in MAS was observed for patients with pre-existing ankle spasticity. Furthermore, TUG decreased by 2.7 s on median, which is a relative TUG change of −24.5%. This indicates a real improvement in balance and mobility on a group-level (Flansbjer et al., 2005). We consider the TUG test as a very important test because it includes many different movements: (i) standing up from a seated position, (ii) walking forward, (iii) turning 180°, (iv) walking back to the chair (v) turning again 180° and (vi) sitting down. Therefore, the TUG shows not only walking speed, but coordination of movements, re-direction of movements and balance. Even-though patients received LE treatment, still an improvement in UE motor function could be observed with an increase of 3.3 points in FMA-UE.

The improvements in walking speed are in line with the ones reported for the experimental BCI groups in literature: 0.13 m/s by Chung et al. (2020) and 0.10 m/s by Mihara et al. (2021). While the observed improvement of 0.19 m/s by Sebastián-Romagosa et al. (2023) is greater than the one observed here, it is important to note that the relative change in walking speed is similar. Again there are some differences in between the current BCI study and the ones of Chung et al. (2020) and Mihara et al. (2021), as they chose unilateral movements. Additionally, Chung et al. (2020) introduced patients to perform motor execution/attempts and Mihara et al. (2021) used fNIRS as recording modality. While fNIRS and EEG allow for similar MI accuracies (Hirsch et al., 2020), fNIRS has inherently lower temporal resolution potentially limiting the users’ experience as the feedback can not be provided synchronously to users’ MI.

In sum, the observed improvement in walking speed of 0.15 m/s is a great result as it equates to walking 90 m farther for every 10 min of walking.

### 4.3 Overall improvement

The most important fact of the current study is that the positive effects of the BCI training did not stop after the first 25 sessions where the UE was treated. In fact, patients progressed further during the LE treatment, and beside the improvements in gait the UE improved as well. Beside all these assessment parameters that we already reported, patients reported many anecdotes that show an improvement in life. Some patients did not need a 4-point walking stick anymore and they changed to 1-point walking sticks or they were able to walk without sticks and even forget the walking sticks in daily life. Other patients used a walker before the therapy and afterwards could walk without it. One patient reported that she is now able to walk downhill and uphill much better and can even care a trolley with her when doing so. Something that was not possible before. Another patient reported that she is now able to play mini-golf, which was impossible before.

In the next study we will investigate the improvement of patients when they perform 50 UE sessions and 50 LE sessions to investigate how much improvement we can get compared to 25 therapy sessions. Another parameter that is interesting to vary is the intensity of the training. In the current study patients are performed 2 or 3 therapies a week. However, a more intense training may be more effective and should thus be investigated (Ballester et al., 2022).

### 4.4 BCI performance

Patients were able to improve their BCI performance during the UE treatment from 73 to 76%, when comparing early and late therapy session (i.e., median first and last five therapy sessions). Importantly, two patients who had a BCI performance of 58 and 68% during the early sessions were able to improve by 14 and 10%, respectively. These results show that stroke patients can learn and improve their MI given enough time and practice.

Patients’ BCI performance was generally greater during the LE compared to the UE treatment. These findings are in line with Neo et al. (2021) who found that crosstalk between hemispheres leads to worse left vs. right hand MI performance in comparison to hand vs. foot MI. Such crosstalk can for example reflect co-activation of ipsi- and contralateral sensorimotor areas during unilateral hand actions (Bai et al., 2005; Serrien et al., 2006; Begliomini et al., 2015). This phenomenon is further exacerbated due to hyperexcitability of the contralesional hemisphere in stroke patients (Dodd et al., 2017), leading to less discriminant features.

Even though patients’ BCI performance was greater during the LE treatment, patients’ BCI performance was nonetheless correlated to the one during the UE treatment. In other words, patients exhibiting greater BCI performance during hand MI also did so for hand vs. foot MI and vice versa. This observation suggests that BCI performance is to some extent intrinsic to the patient, even though improvements are possible, as described previously. While there are predictors of MI performance based on resting state EEG such as theta and mu power (Ahn et al., 2013), spectral entropy (Zhang et al., 2015), microstates (Cui et al., 2023) and connectivity (Lee et al., 2020), these analyses are typically specific to healthy individuals necessitating similar studies in stroke patients.

One limitation of the current BCI performance analysis is that patients were not in a cross-over study design. Therefore, it is difficult to determine why patients were able to improve their BCI performance during UE but not LE treatment. Here we propose two possible explanations: (i) In the current study patients learned MI during the first UE treatments, which lead to the observed improvements in BCI performance during the UE treatment. However, once patients learned MI their BCI performance stayed reasonable stable. (ii) Hand vs. foot MI may be an inherently easier task compared to hand MI, thus patients’ improvements in BCI performance occur already early on within the first therapy sessions.

Most importantly the BCI classification accuracy was in both cases above the 62% significance thresholds for the 240 trials (i.e., movement repetitions) in one session. In order to control the FES device and the avatar with a meaningful accuracy, patients are supposed to be above this threshold. Interestingly, our stroke patients’ classification accuracy (i.e., maximum accuracy during feedback phase) is comparable to the one obtained with students in a study carried out by Ortner et al. (2015). Specifically, Ortner et al. (2015) found an average classification accuracy of 81% in a group of 20 students for performing left and right hand MI. Back then we thought it will be difficult for stroke patients to reach this level. However, here our stroke patients reached 79 and 85% classification accuracy on average for hand and hand vs. foot MI, respectively.

### 4.5 Future work

The current study does have some limitations which need to be acknowledged and should be addressed in future work.

The improvements after the LE BCI training are similar to the ones reported for LE BCIs in literature. However, the current study did not employ a separate LE BCI group as control group, which would allow to investigate how improvements achieved by patients who underwent UE followed be LE BCI training compares to patients who only underwent LE BCI training using the same BCI system.

While the studied patient sample is relatively heterogenous with respect to stroke type and lesion location, it is nonetheless small in size. Greater and more nuanced insights with respect to the observed improvements, as well as the relationship of stroke type and lesion location to the BCI system’s efficacy, as well as patients’ BCI performance can only be investigated in larger patient populations.

As described by Sebastián-Romagosa et al. (2020b) BCI therapies allow for the unique opportunity to investigate patients’ brain activity during and across the treatment. Thus, group-level and subject-level changes in brain activity and their relationship to the functional state can be studied and monitored. Additionally, changes in patients’ brain activity can be investigated to gain further insights in how neuroplasticity facilitated by BCI training drive the functional improvements, observed.

## 5 Conclusion

In the current study 19 stroke patients trained with a BCI system based on MI, FES and a realistic 3D avatar across 25 upper followed by 25 lower extremity therapy sessions. The BCI system rewarded active participation and correct MI in real-time by providing proprioceptive and visual feedback using FES and a realistic 3D avatar. Results of the current work show that patients improve in the targeted extremities and activities of daily living. Following the upper extremity BCI treatment, significant improvements in upper extremity motor function, activities of daily living, as well as clinically relevant improvement in wrist and finger spasticity were observed. Furthermore, the same patients showed continued improvements during the second BCI treatment, as clinically relevant improvements in ankle spasticity, mobility, and balance, as well as walking speed were observed. Taken together, this study provides evidence that patients undergoing upper extremity BCI treatment derive additional benefits from subsequent lower extremity BCI treatment.

## Data availability statement

The datasets presented in this article are not readily available because patients’ data need to be treated according to current data protection laws and ethical guidelines. Requests to access the datasets should be directed to CG, guger@gtec.at.

## Ethics statement

The studies involving humans were approved by Ethikkommission des Landes Oberösterreich, Austria (Nr. 1,126/2020 and #D-42-17) and the Bundesamt für Sicherheit im Gesundheitswesen (clinical trial number 101210314). The studies were conducted in accordance with the local legislation and institutional requirements. The participants provided their written informed consent to participate in this study.

## Author contributions

SS: Data curation, Formal analysis, Investigation, Methodology, Visualization, Writing – original draft. MS-R: Data curation, Investigation, Methodology, Supervision, Writing – review & editing. WC: Data curation, Investigation, Supervision, Writing – review & editing. JG: Supervision, Writing – review & editing. RO: Supervision, Writing – review & editing. JS: Supervision, Writing – review & editing. KK: Supervision, Writing – review & editing. CG: Supervision, Writing – review & editing.
